# Predicting Challenge and Threat Appraisal of Job Demands among Nurses: The Role of Matching Job Resources

**DOI:** 10.3390/ijerph20021288

**Published:** 2023-01-11

**Authors:** Martha Fernandez de Henestrosa, Philipp E. Sischka, Georges Steffgen

**Affiliations:** Department of Behavioural and Cognitive Sciences, University of Luxembourg, L-4366 Esch-sur-Alzette, Luxembourg

**Keywords:** job demands, challenge appraisal, threat appraisal, job resources, matching principle

## Abstract

(1) Background: Empirical studies have started to examine employees’ subjective appraisals of job demands and their relations to employees’ health. However, knowledge of working conditions, which might contribute to how employees appraise specific job demands, is scarce. The present study aimed to examine predictors of nurses’ appraisals of job demands (i.e., time pressure, emotional demands, physical demands, and role ambiguity) as challenges and/or threats among corresponding job resources (i.e., autonomy, social support, physical resources, participation in decision-making). It also examined moderating effects of these predictors. (2) Methods: Cross-sectional data were collected via an online survey in a sample of 425 nurses working in Luxembourg. (3) Results: Multiple regression analyses indicated that matching job resources predicted nurses’ appraisal of job demands as challenging. Threat appraisal was predicted by three out of four kinds of job resources (i.e., autonomy, physical resources, participation in decision-making). However, the current study did not find any moderating effects between job demands and job resources on challenge/threat appraisals. (4) Conclusions: The present study identified domain-specific job resources that contribute to how employees perceive selected job demands. Accordingly, we encourage scholars and practitioners to align job demands with matching job resources to prevent nurses’ threat appraisal of job demands, and to promote their challenge appraisals.

## 1. Introduction

Up to now, research on the topic of workplace stress has shown that work-related stressors, henceforth referred to as job demands, play a vital role with respect to employees’ health and well-being [1]. According to the Job Demands–Resources (JD–R) model, job demands are defined as aspects of work, which demand effort and associate with physiological/psychological costs [1]. Notably, most of the studies conducted in this research area have shown that job demands (e.g., work pressure, emotional demanding interactions) tend to relate to ill-being outcomes (e.g., exhaustion, burnout) [1]. However, subsequent research has also demonstrated that some job demands might positively impact employees’ well-being (i.e., increased motivation, work-engagement) and performance [2,3,4]. Accordingly, scholars have so far identified, examined, and categorized different types of job demands (i.e., challenge, hindrance and/or threat) based on their associations with health-, motivation-, and performance-related outcomes [3].

Note, however, that recent studies on the topic of work-related stressors have moved beyond the mere use of a priori categorization of job demands to explain inconsistent findings between work-related stressors and diverse outcomes of interest [3]. Specifically, attention has been placed on employees’ subjective evaluations (i.e., appraisals) of job demands and how these perceptions affect the well-being, growth, and performance of workers [3,5,6,7]. For instance, research has shown that employees’ subjective appraisals of job demands relate to strain, attitudes, and behavioral intensions [7]. However, little is in known on the question of whether there are work-related factors, which might contribute to how employees understand and perceive specific job demands [3,8]. Which organizational properties might serve as precursors of employees’ demand appraisals? Which work-related boundary conditions might impact employees’ appraisal of these demands? Therefore, the present study seeks (a) to examine predictors of employees’ demand appraisals, and (b) to investigate potential moderating effects of these predictors. Identifying situational factors, which contribute to how employees understand and experience certain job stressors, might help us to manage and navigate the effects of job demands on work-related outcomes [3,9].

### 1.1. Examining Subjective Appraisal of Job Demands

Drawing on the Transactional Theory of Stress (TTS) [10], a growing number of studies have begun to examine employees’ subjective appraisals of job demands as mechanisms, which may transmit the effects of job stressors on various work-related outcomes [3]. Subjective appraisal is defined as the individuals’ evaluation of a specific situation (or stressor) with respect to his/her own well-being, and can be further differentiated into challenge (i.e., anticipated mastery and gain) and threat appraisal (i.e., anticipated harm and loss) [10,11]. Note that previous research has empirically confirmed that job demands and the appraisal of these demands are two separate constructs [5]. Whereas “job demands are defined as those physical, psychological, social, or organizational aspects of the job that require sustained physical and/or psychological effort and are therefore associated with certain physiological and/or psychological costs” [1] (p. 274), appraisal refers to employees’ perceptions of these job stressors [12]. Accordingly, it has been argued that such appraisal processes might mediate the effects of job demands on work-related outcomes [13]. Indeed, it has been shown that the associations between job demands and well-being outcomes are mediated, in part, by employees’ appraisals of job demands [5,7]. Moreover, subjective appraisal has been found to significantly relate to employees’ coping strategies, affective and behavioral responses, as well as to employees’ working attitudes and performance [5,7]. In addition, recent research has shown that challenge and threat perceptions relate to psychological distress, becoming central variables as regards to the prediction of mental health [14]. Finally, and in line with the TTS, previous studies have shown that employees might appraise job demands simultaneously as having the potential for harm and gain, and that these appraisals may fluctuate from day to day [5,7].

Although this stream of research has extended our understanding on the nature and functioning of job demands, knowledge on work-related factors, which might contribute to how employees appraise specific job demands, is scarce [3,8]. However, understanding which factors predict employees’ appraisal of work-related stressor as challenging and/or threatening is of theoretical and practical importance [3]. From a theoretical perspective, it may serve to revise and expand existing frameworks and models of workplace stress, and consequently, to accumulate knowledge in this field of research [3]. From a practical perspective, it may inform and guide the development of workplace interventions directed at promoting employees’ challenge appraisal and reducing their appraisal of job demands as threatening [8]. Therefore, the present study seeks to expand research on the topic of workplace stressors by examining determinants of challenge and threat appraisals among matching job resources.

### 1.2. Job Resources as Determinants of Challenge and Threat Appraisals of Job Demands

We drew from three theoretical frameworks to examine predictors of challenge and threat appraisals of job demands: the Transactional Theory of Stress (TTS) [10], the Job Demands–Resources model (JD–R) [1], and the Demand-Induced Strain Compensation model (DISC) [15]. First, and in line with the TTS, we argue that it is important to consider situation-related factors when examining possible predictors of individuals’ appraisals. According to this theory, contextual factors contribute to the development of challenge and threat appraisals [10]. Examples of such factors are ambiguity and event uncertainty, which have been theorized to intensify the likelihood of a stressor being appraised as threatening [10]. In this regard, ambiguity refers to a lack of situational clarity (i.e., lack of information about the situation), whereas event uncertainty implies that characteristics of the environment are not predictable, and therefore cannot be distinguished, noticed, or learned [10]. However, the notion of predictability is crucial, as it implies having control over the environment [10]. By transferring this knowledge to a work context, scholars have argued that organizations can reduce uncertainty and lack of clarity by providing employees with information and opportunities for control over their work environment, consequently influencing how employees appraise job demands [13].

Second, we note that the above-mentioned dimensions of control and provision of information are reflected within specific elements of the JD–R model. Specifically, they constitute prominent examples of job resources [9]. According to the JD–R model, job resources are defined as “physical, psychological, social, or organizational aspects of the job that are functional in achieving work goals, reduce job demands and the associated physiological and psychological costs, or stimulate personal growth, learning, and development” [1] (p. 274). Job resources are pivotal characteristics of the job, as they yield positive effects for employees and organizations [1]. To give an illustration, work-related resources have been found to predict employees’ well-being, job attitudes and performance [1]. That said, job resources might not only help employees to weaken the negative effects of job demands [1], but might also influence how employees perceive the demands themselves [9]. In fact, there is growing evidence for the functioning of job resources as determinants of demand appraisals. To give an illustration, a study examining the role of participative climate (i.e., job resource) in the relation between work intensification (i.e., job demand) and cognitive appraisal found that a favorable participative climate was linked to employees appraising the intensification of work as less hindering [13]. A cross-lagged study conducted among US Marines has shown that charismatic leadership (i.e., job resource) influences the appraisal of stressors as challenging [16]. Likewise, a cross-sectional study found that job control and social support significantly related to employees’ appraisal of certain job demands (e.g., time pressure, task complexity, responsibility, interruptions) [9]. Finally, a recent cross-sectional study identified vertical and horizontal trust at work (i.e., job resources) to predict the appraisal of job demands [8]. However, research on situational determinants of appraisal at work has just begun, with more research needed to understand the role of work-related factors (i.e., job resources) with respect to employees’ appraisal process [3].

Third, we argue that it is important to consider corresponding job resources when examining predictors of demand appraisal. Specifically, previous theoretical advancements in research on job stress among human service occupations (e.g., nursing) have proposed that the functioning and interaction of job demands and job resources (and well-being outcomes) might be strongest when the involved concepts derive from the same qualitative domain (i.e., matching principle) [15]. In particular, and according to the DISC model, we may distinguish between cognitive, emotional, and behavioral (physical) domains [15]. As such, cognitive demands are more inclined to interact with cognitive resources on cognitive kinds of strain, emotional demands are more inclined to interact with emotional resources on emotional kinds of strain, and physical demands are more inclined to interact with physical resources on physical kinds of strain [15]. For instance, suppose an employee experiences emotional demanding interactions at work (e.g., being confronted with a complaining patient), which results in negative emotions [15]. According to this model, emotional resources, such as the support provided by a colleague (e.g., providing sympathy/re-assurance), might best counteract the negative emotions experienced by the employee [15]. Similarly, if an employee experiences time pressure at work, he/she might best use his/her decision autonomy to reorganize, or even hold up, certain tasks [15]. That is, job resources should match job demands [15]. Drawing on this principle, the current study aimed to advance knowledge on work-related factors that predict employees’ demand appraisal by acknowledging the specificity of the underlying qualitative domain so that the job resources under consideration correspond to the job demands at stake.

### 1.3. The Present Study

The aims of the current study were twofold. The first aim was to identify predictors of nurses’ challenge and threat appraisals of job demands among matching job resources. In particular, and due to their person-centered job, nurses are generally at an increased risk of experiencing strain at work [17]. Hence, we were interested to examine factors that might affect how nurses appraise work-related stressors. The current study focused on time pressure and role ambiguity (i.e., work-related stressors corresponding to the cognitive dimension), emotional demands (i.e., work-related stressor corresponding to the emotional dimension), and physical demands (i.e., work-related stressor corresponding to the physical dimension). These job demands were chosen, as (a) they reflect qualitative different domains (i.e., cognitive, emotional, physical) [17], and (b) apply to the nursing occupation [17,18,19,20,21].

With respect to the selected job resources, the current study focused on autonomy and participation in decision-making (i.e., work-related resources corresponding to the cognitive dimension), social support (i.e., work-related resource corresponding to the emotional dimension), and physical resources (i.e., work-related resource corresponding to the physical dimension) [17]. As with the above-mentioned job demands, we chose the present resources as they represent key job resources of nursing staff [19,20,22]. Moreover, these resources are important contextual factors that have been included in previous theoretical frameworks on job stress and are considered to be functional in dealing with job demands [1,15,17]. For instance, autonomy and participation in decision-making refer to the notion of control at work [1,9]. In fact, both resources have the potential to boost employees’ control over the work environment, to decrease lack of clarity, and consequently, to positively influence the appraisal of encountered job demands [13]. Note at this point that autonomy refers to employees’ control as regards to the execution of tasks [23], whereas participation in decision making implies some degree of influence at work (i.e., shared decisions), which in turn might allow employees to cope with job demands [8,13]. Another prominent job resource is social support [24]. It is one of the most examined work-related resources in studies conducted on the JD–R model [25]. In this regard, studies have shown that social support relates to health, job satisfaction, work-engagement, and performance [26,27]. Lastly, physical resources, such as ergonomic aids, also play a role in dealing with (physical) job demands [17]. In fact, physical resources have been found to buffer the adverse effects of (physical) demands on employees’ well-being [17]. For an overview of the connections between our studied variables and their theoretical frameworks, please refer to Table 1.

Drawing on previous studies examining the role of job resources as predictors of demand appraisal [8,13], and bearing in mind the tenants of the DISC model [15], we believe that corresponding job resources will predict the appraisal of job demands. Based on these theoretical considerations and previous empirical findings, we formulated eight hypotheses. The first four hypotheses address the link between work-related resources and the appraisal of work-related demands as challenging. That is, we expected that employees reporting higher levels of job resources would appraise matching job demands as more challenging.

**Hypothesis** **1a.** *Autonomy positively relates to the appraisal of time pressure as challenging.*

**Hypothesis** **1b.** *Social support positively relates to the appraisal of emotional demands as challenging.*

**Hypothesis** **1c.** *Physical resources positively relate to the appraisal of physical demands as challenging.*

**Hypothesis** **1d.** *Participation in decision-making positively relates to the appraisal of role ambiguity as challenging.*

The remaining four hypotheses address the link between work-related resources and the appraisal of work-related demands as threatening. That is, we expected that employees reporting higher levels of job resources would appraise matching job demands as less threatening.

**Hypothesis** **2a.** *Autonomy negatively relates to the appraisal of time pressure as threatening.*

**Hypothesis** **2b.** *Social support negatively relates to the appraisal of emotional demands as threatening.*

**Hypothesis** **2c.** *Physical resources negatively relate to the appraisal of physical demands as threatening.*

**Hypothesis** **2d.** *Participation in decision-making negatively relates to the appraisal of role ambiguity as threatening.*

The second aim was to contribute to an enhanced understanding regarding work-related conditions under which job demands function as challenges and/or threats by examining the above-mentioned domain-specific job resources as potential moderators of the demand–appraisal relationship. Drawing on previous theoretical developments [10], we would expect that (work-related) resources may affect the association between (work-related) stressors and appraisals in a positive manner. First, studies have provided support for this assumption. For instance, scholars have shown that job resources such as a favorable participative climate weaken the relation between work intensification (i.e., job demand) and the negative appraisal of the demand [13]. In a similar vein, job control moderated the relation between certain job demands (i.e., task complexity, interruptions, responsibility) and their respective hindrance appraisals [9]. In addition, a cross-lagged study showed that, under conditions of high charismatic leadership (i.e., job resource), challenge stressors associated more positively with challenge appraisals [16]. Therefore, and drawing on the previously described matching principle [15], we expected that domain-specific job resources would moderate the relation between matching job demands and the appraisal of these demands (challenge, threat).

**Hypothesis** **3a.** *Autonomy moderates the relation between time pressure and the appraisal of time pressure (i) as challenging ((ii) as threatening), in that the relation is strengthened (weakened) for high levels of autonomy.*

**Hypothesis** **3b.** *Social support moderates the relation between emotional demands and the appraisal of emotional demands (i) as challenging ((ii) as threatening), in that the relation is strengthened (weakened) for high levels of social support.*

**Hypothesis** **3c.** *Physical resources moderate the relation between physical demands and the appraisal of physical demands (i) as challenging ((ii) as threatening), in that the relation is strengthened (weakened) for high levels of physical resources.*

**Hypothesis** **3d.** *Participation in decision-making moderates the relation between role ambiguity and the appraisal of role ambiguity (i) as challenging ((ii) as threatening), in that the relation is strengthened (weakened) for high levels of participation in decision- making.*

## 2. Materials and Methods

### 2.1. Data Collection

The current study was carried out as part of a larger research project on nurses’ working conditions in Luxembourg. The University of Luxembourg and the Association Nationale des Infirmières et Infirmiers du Luxembourg jointly implemented the project. The study followed the guidelines of the Declaration of Helsinki and informed consent was obtained from all participants. The Ethics Review Panel of the University of Luxembourg formally approved the research project. Data were collected via an online survey in 2021. Note at this point that data gathering took place during the COVID-19 pandemic. Participants could reply to the survey in French or in German. All data reported in the present study are cross-sectional.

### 2.2. Participants

Inclusion criteria were the following: (a) working at least 20 h/week, (b) executing for at least 6 months a nursing profession [28]. Nurses working in Luxembourg were recruited via flyers, newsletters, posts on social media, as well as via radio and TV reportages informing about the project. In the context of the present study, convenience sampling was used. A total of 1062 clicks on the survey link were registered. We removed 250 cases in which no responses were recorded (i.e., blank) and excluded 77 cases, as they did not meet the inclusion criteria, which resulted in a sample of 735 participants. However, the survey was characterized by a high dropout rate, and only 425 participants filled in the complete questionnaire (The COVID-19 pandemic constitutes a rather difficult time of data collection. Nurses were exposed to increased stress during this time, which may in turn explain the increased dropout rate). Thus, the effective sample consisted of 425 nurses working in Luxembourg (75% female, *n* = 318). Residents from Luxembourg (62.6%, *n* = 266), as well as commuters from France (18.1%, *n* = 77), Germany (9.9%, *n* = 42), and Belgium (9.2%, *n* = 39), were included. The nurses’ ages ranged from 21 to 61 years (*M* = 40.17, *SD* = 9.89). Nurses indicated working, on average, 35.48 contractual hours per week (*SD* = 5.92). Their average experience in nursing was 190.37 months (*SD* =129.02), which is about 16 years. Most nurses indicated working in hospitals (73.6%, *n* = 313), followed by 13.6% of nurses working in nursing homes/social sector (*n* = 58), and 12.7% of nurses (*n* = 54) indicated working in other areas (e.g., government agencies, associations, etc.). A total of 95% of nurses (*n* = 402) reported having a permanent work contract compared to 5% (*n* = 21) of nurses with a temporary contract. Nurses indicated to have, on average, 6.95 days per month (*SD* = 5.50) of atypical working hours (i.e., working at night or on the weekends). Overall, 53.2% filled in the survey in German (*n* = 226), and 46.8% filled in the survey in French (*n* = 199).

### 2.3. Measures

We used consistently validated and proven scales to measure the relevant variables. In cases where not all required language versions were available, translations of the scales were created. The first author and a research assistant translated the measures, which were not yet in French and/or German. Afterwards, both colleagues decided, through dialogue, on a shared translation of the measures (i.e., committee-based approach) [29,30]. Subsequently, the French/German translations were back-translated by two bilingual experts (i.e., back-translation approach) [29]. Note that the internal consistencies for all the scales are displayed in Section 3.

#### 2.3.1. Job Demands

We used scales from the Quality of Work Index (QoW) to measure *time pressure* (e.g., “How often are you required to meet tight deadlines in your work?”), *emotional demands* (e.g., “How often does your work require you to control your feelings?”), and *role ambiguity* (e.g., “To what extent are your work tasks clearly defined?”) [31]. (We reversed the two items to represent role ambiguity). The three scales consisted of two items, respectively. Participants had to indicate their answers on a 5-point Likert response scale (for time pressure and emotional demands: 1 (= *never*) to 5 (= *always*); for role ambiguity: 1 (= *to a very low extent*) to 5 (= *to a very large extent*)). We assessed *physical demands* with the DISC Questionnaire (DISQ-S 3.0) [32,33,34,35].(We employed the validated French translation of the DISQ version 2.0 as baseline for adaptations, as we did not find a previously validated French translation of the DISQ-S version 3.0. We marginally adapted the French wording of the items to match the wording of the DISQ-S version 3.0 (English version/German version)) A total of three items represented the dimension of *physical demands* (e.g., “I have to perform a lot of physically strenuous tasks to carry out my job”). Again, participants had to indicate their answers on a 5-point Likert response scale ranging from 1 (= *never*) to 5 (= *always*).

#### 2.3.2. Job Resources

To measure three of the four job resources, we used scales from the QoW-Index [31]. Specifically, the scale *autonomy* consisted of four items (e.g., “To what extent can you decide how you carry out your work?”), whereas the scale *social support* consisted of three items (e.g., “To what extent are you supported in your work by your colleagues?”). The scale *participation in decision-making* consisted of two items (e.g., “To what extent are you involved in decisions in your organization?”). Participants were indicated to answer on a 5-point Likert scale, ranging from 1 (= *to a very low extent*) to 5 (= *to a very large extent*). *Physical resources* were assessed with the DISQ-S 3.0 [32,33,34,35] (We employed the validated French translation of the DISQ version 2.0 as baseline for adaptations, as we did not find a previously validated French translation of the DISQ-S version 3.0. We marginally adapted the French wording of the items to match the wording of the DISQ-S version 3.0 (English version/German version)). The scale consisted of three items (e.g., “I am able to use adequate technical equipment to accomplish physically strenuous tasks”). Participants had to indicate their answers on a 5-point Likert response scale ranging from 1 (= *never*) to 5 (= *always*).

#### 2.3.3. Appraisal

We used a four-item scale to measure challenge appraisal (e.g., “…will make the experience educational”) [5]. (We slightly modified the English wording of two items based on recent research [6,36,37]). Drawing on previous research [6], we used a three-item scale to measure threat appraisal (e.g., “…is going to have a negative impact on me”) [38]. For each of the four job demands, participants were asked to indicate on a 5-point Likert scale (1 = *not at all* to 5 = *completely*) to what extent they perceived a given job demand to be a challenge, as well as to be a threat.

### 2.4. Data Analysis

We performed statistical analyses using R version 4.0.5 [39]. (The R code for all analyses is available in the Supplemental Material). In a first step, we performed confirmatory factor analyses to test the measurement structure of the study variables. Considering the distributional nature of the data, we applied a maximum likelihood estimation with robust standard errors and scales test statistics (MLR) [40]. We used the marker variable method for scale setting [41]. To assess the fit of the hypothesized measurement models, we considered commonly reported fit statistics, namely χ2 with its degrees of freedom and *p* value, the Root Mean Square Error of Approximation (RMSEA), the Standardized Root Mean Square Residuals (SRMR), the Comparative Fit Index (CFI), and the Tucker–Lewis Index (TLI) [42,43,44]. Concerning the RMSEA, values up to 0.06 (with 90% confidence interval of 0.00–0.08) indicate a good fit, and for the SRMR, values of less than 0.08 indicate a good fit [43]. Regarding the CFI and the TFI, values of 0.95 signal a good fit [41,43]. In the second step, we examined reliability, means, standard deviations, and zero-order correlations between the study variables. Note that we used McDonald’s omega (ω) instead of Cronbach’s alpha to estimate the internal consistency of the scales, as omega is a more general estimator of reliability that relies on a less strict measurement model than alpha [45]. In a third step, we conducted regression analyses to analyze the associations between our hypothesized predictor variables (i.e., job demands and job resources) and our criterion variables (i.e., challenge and threat appraisal). Specifically, we performed simple moderation analysis to (a) examine the main effects of job demands and matching job resources on nurses’ challenge and threat appraisal of job demands, as well as to (b) examine possible interaction effects of job demands and job resources on demand appraisals. For each set of regression analysis, predictor variables were centered prior to analysis. Due to multiple testing, we applied the Bonferroni correction to tailor the α-value, and therefore, to control for inflated Type I error [46]. Considering that we focused on two criterion variables, we divided the α-value (.05) by the number two. Consequently, the criterion for significance was set to *p* < 0.025.

## 3. Results

### 3.1. Factor Analysis

We performed confirmatory factor analyses (CFA) to test the measurement structure of the study variables. We developed four separate measurement models, one for each job demand and its respective appraisals: (a) time pressure, (b) emotional demands, (c) physical demands, and (d) role ambiguity. Note that each measurement model included the job resource, which matched the specific job demand (e.g., for the model of time pressure, we included autonomy). To prevent estimation problems, we equated the item factor loadings of latent variables consisting of only two items (This technique is based on the assumption that the items are equivalent in terms of their loadings, but may differ regarding their intercepts (i.e., tau-equivalent indicators) [41]). Table 2 summarizes the results. The fit statistics of the four measurement models showed a good fit. Item loadings ranged from 0.50 to 0.93 and were significant at *p* < 0.001. These results support the predicted measurement model of the assessed constructs. We have added figures illustrating the measurement structure of the study variables on OSF (See OSF link: https://doi.org/10.17605/OSF.IO/WHZJ9, accessed on 6 January 2023). In addition, we investigated measurement invariance between both language versions. Specifically, we examined the factorial structure of the chosen job demands, job resources, and challenge/threat appraisals for the German and for the French version of the questionnaire via multi-group confirmatory factor analyses (MG-CFA). Evidence for partial scalar invariance was found, which in turn suggests that both language versions are appropriate to be used for further analysis. Findings from these preliminary analyses can be found on OSF. (See OSF link: https://doi.org/10.17605/OSF.IO/WHZJ9, accessed on 6 January 2023).

### 3.2. Descriptive Statistics and Correlational Analysis

Table 3 Shows means, standard deviations, reliabilities (McDonald’s Omega), and inter-correlations between the study variables. In terms of reliability, values ranged between 0.69 and 0.93, suggesting moderate to high reliability. The data were slightly skewed (univariate skewness ranged between 0.02 and 1.04) and kurtotic (univariate kurtosis ranged between −0.86 and 1.35), but below the absolute values of 2 and 7, respectively [43]. Multicollinearity was negligible, as all correlations were way below 0.80. All job demands correlated significantly and positively with each other, except for the correlation between physical demands and role ambiguity (*p* > 0.05). Likewise, all job resources correlated significantly and positively with each other. In addition, job demands (i.e., time pressure, emotional demands, and role ambiguity) correlated negatively with their respective challenge appraisals, and positively with their respective threat appraisals. As regards to the correlations between job demands and matching job resources, note that each job resource (i.e., autonomy, social support, physical resources, participation in decision making) significantly correlated with its matching job demand (i.e., time pressure, emotional demands, physical demands, role ambiguity), as well as with the respective challenge/threat appraisal of that demand.

### 3.3. Multiple Regression Analyses

To test the hypothesis 1 (a–d), 2 (a–d), and 3 (a–d), a total of eight multiple regression analyses were conducted with challenge appraisal and threat appraisal as the criterion variables. That is, for each criterion variable, we conducted four simple moderation analyses—one for each job demand and matching job resource.

As can be seen in Table 4, Table 5, Table 6 and Table 7, all job resources significantly predicted the appraisal of job demands as challenging. More specifically, autonomy was positively related to the appraisal of time pressure as challenging (β = 0.12, *p* = 0.021). Likewise, social support was positively related to the appraisal of emotional demands as challenging (β = 0.13, *p* = 0.011). In addition, physical resources positively related to nurses’ challenge appraisal of physical demands (β = 0.21, *p* < 0.001), and participation in decision-making positively related to the appraisal of role ambiguity as challenging (β = 0.14, *p* = 0.007). These findings support hypothesis 1 (a–d). Thus, nurses reporting higher levels of job resources appraised job demands as more challenging. In addition, role ambiguity is significantly related to challenge appraisal (β = −0.16, *p* = 0.002). However, no interaction effects emerged between job demands and matching job resources on challenge appraisal (i.e., H3a–d; all *p* > 0.05).

As regards the prediction of nurses’ threat appraisal of job demands, autonomy, physical resources, and participation in decision-making were identified as significant predictors. Specifically, autonomy negatively related to the appraisal of time pressure as threatening (β = −0.12, *p* = 0.016). Similarly, physical resources negatively related to the appraisal of physical demands as threatening (β = −0.14, *p* = 0.002), whereas participation in decision-making negatively related to the appraisal of role ambiguity as threatening (β = −0.21, *p* < 0.001). However, social support did not significantly relate to the appraisal of emotional demands as threatening (β = −0.07, *p* = 0.180). These findings provide partial support of hypothesis 2 (a–d). That is, nurses reporting higher levels of autonomy, physical resources, and participation in decision-making appraise matching job demands as less threatening. In contrast, appraising emotional demands as threatening was unrelated to nurses’ experience of social support. In addition, time pressure (β = 0.36, *p* = 0.001), emotional demands (β = 0.31, *p* < 0.001), physical demands (β = 0.38, *p* < 0.001), and role ambiguity (β = 0.19, *p* < 0.001) significantly predicted threat appraisal. However, no interaction effects emerged between job demands and matching job resources on threat appraisal (i.e., H3a–d; all *p* > 0.05).

## 4. Discussion

The present study aimed to investigate predictors of employees’ challenge and threat appraisals of job demands among domain-specific job resources, as well as to examine possible moderating effects of these predictors. We focused on four job demands (i.e., time pressure, emotional demands, physical demands, and role ambiguity), four job resources (i.e., autonomy, social support, physical resources, and participation in decision-making), and their associations to challenge and threat appraisals. We analyzed (a) whether matching job resources predict the appraisal of job demands as challenging/threatening, and (b) whether corresponding job resources may act as moderators of the demand–appraisal relationship.

Overall, results of the current study affirmed that work-related resources are associated with nurses’ subjective appraisal of work-related demands. All four job resources predicted challenge appraisals, so that nurses reporting higher levels of resources appraised matching job demands as more challenging. That is, the more autonomy, social support, physical resources, and participation in decision-making nurses reported to experience, the more they perceived time pressure, emotional demands, physical demands, and role ambiguity as challenging. Concerning nurses’ threat appraisals, results slightly differed. According to hypothesis 2 (a–d), we assumed that all domain-specific job resources would negatively relate to nurses’ threat appraisals of work-related demands. However, not all job resources were identified as significant predictors. That is, three out of four job resources were found to predict nurses’ threat appraisals. Specifically, the more autonomy, physical resources, and participation in decision-making nurses reported to experience, the less they perceived corresponding job demands, such as time pressure, physical demands, and role ambiguity, as threatening. However, nurses’ threat appraisal of emotional demands was unrelated to the level of social support they experienced. Taken together, these findings suggest that, while social support might act as an important predictor of nurses’ appraisal of emotional demands as challenging, it does not relate to their perception of this demand as threatening. Lastly, and in contrast to our hypothesis 3 (a–d), the current study did not find any evidence for possible moderating effects of job resources on the relation between job demands and appraisals. Thus, rather than serving as a work-related boundary condition contributing to individuals’ appraisals of job demands, the current study showed that matching job resources may instead act as predictors of nurses’ challenge and threat appraisals. That is, matching job resources may act as protective factors with respect to nurses’ challenge and threat appraisals. In summary, findings of the current study are largely in line with previous research examining the associations between resources at work and employees’ appraisals [8], and with the transactional theory of stress, which corroborates the importance of situational factors in view of individuals’ appraisals of (work-related) stressors [10]. Most notably, our findings also support the DISC model [15], in that the chosen types of resources examined in the current study match the job demands at stake. Thus, the present findings suggest that considering domain-specific job resources that coincide with job demands might help us to get a more refined understanding on the nature and functioning of job demands, and more specifically on how employees understand and experience the corresponding demand.

### 4.1. Theoretical Contributions

Results of the current study contribute to the literature and existing theoretical frameworks on the topic of workplace stress as follows. First, the present study addresses the recent call to examine contextual factors, which might contribute to how employees appraise specific job demands [3]. In doing so, we combined multiple theoretical models, seeking to accumulate knowledge in this field of research. In particular, and drawing on the TTS [10], the JD–R model [1], and the DISC model [15], the current study examined predictors of challenge and threat appraisals among specific job resources. Second, the present study expands previous research on situational factors that may influence stress appraisal [8,9,13,16], by considering the matching principle [15], examining the appraisal of domain-specific job demands alongside job resources of the same kind. Accordingly, results of the present study corroborate the significance of considering matching job resources to predict the appraisal of work-related demands as challenging and threatening. Therefore, the findings of the current study might serve to revise and expand knowledge on the nature and functioning of job demands.

### 4.2. Practical Implications

Results of the current study highlight the importance of job resources with respect to how employees perceive and evaluate work-related demands. As findings have shown, matching job resources predicted nurses’ subjective appraisal of job demands. Hence, from a practical perspective, organizational interventions should consider domain-specific job resources that contribute to nurses’ challenge and threat appraisals of job demands. In other words, we encourage practitioners to consider job resources that correspond to the type of job demands when planning and developing measures used in workplace health promotion. Specifically, employers should promote job resources, such as autonomy, physical resources, and participation in decision-making to prevent nurses’ threat appraisal of matching job demands (i.e., time pressure, physical demands, role ambiguity), as well as promoting their challenge appraisals. This, in turn, might be a starting point for dealing with the effects of job demands on employees’ well-being [3].

### 4.3. Limitations and Future Research

Although the current study contributes to research on the nature and functioning of job demands and associated appraisals, some limitations need to be addressed. First, the present study used a cross-sectional design, which precludes conclusions about causality. However, it should be noted that our aim was not to infer cause and effect relationships, but to investigate (predictive) associations between work-related demands and resources with regards to employees’ subjective appraisals. Notwithstanding, scholars might further examine predictors of challenge and threat appraisals using longitudinal and (quasi-) experimental research designs. Second, results are based on self-report measures, which might be affected by common-method bias. However, previous research conducted on the appraisal of job demands has commonly used self-report measures [5]. Indeed, we believe that the use of objective methods (e.g., observer ratings) would have been inappropriate given the subjective nature or our constructs of interest (i.e., challenge and threat appraisals). Third, as the present study used a convenience sampling approach, a potential selection bias cannot be excluded. Thus, the study sample might not be representative for the nursing population in Luxembourg. Fourth, we focused exclusively on situational/contextual factors (i.e., job resources) as predictors of employees’ challenge and threat appraisals. However, other factors, such as individual attributes, might contribute to employees’ appraisals of work-related demands [3]. In recent research, scholars have outlined the merits of including personal resources, such as resilience, into the examination of protective factors of strain outcomes (e.g., burnout) among health care professionals [19]. Therefore, future studies could expand the findings of the present study by examining individual attributes, such as resilience, as predictors (and possible moderators) of challenge/threat appraisals. Fifth, we did not include sociodemographic variables into the regression/moderation analyses, as we had no specific hypotheses about the relationships between the demographic variables and challenge/threat appraisal. Moreover, and considering the recent debate about control variables [47,48,49,50], we refrained from including them in our regression models. However, future studies might examine the association between different demographic variables and challenge/threat appraisals. In a similar vein, scholars might also consider exploring possible gender differences regarding the experience and perception of encountered demands and resources at work. Indeed, recent research on work characteristics found that female and male workers differ in regard to the reported levels of certain job characteristics (e.g., physical demands, equipment use) [51]. Finally, note that due to the COVID-19 pandemic, nurses may have experienced increased levels of job stress, which in turn might have affected the intensity of the relationship between job resources and the appraisal of job demands as challenging/threatening. Therefore, future studies might examine the resource–appraisal associations beyond pandemic times.

## 5. Conclusions

The current study examined matching job resources as predictors of nurses’ challenge and threat appraisals of job demands and investigated moderating effects of these predictors. Results demonstrated that job resources predict the subjective appraisal of corresponding job demands as challenging/threatening. However, no evidence was found for moderating effects. To conclude, the present study identified important resources at work, which require special attention to promote nurses’ challenge appraisal, and to preclude their threat appraisal. Accordingly, we encourage scholars and practitioners to match job demands with corresponding job resources when designing and carrying out organizational interventions.

## Figures and Tables

**Table 1:** Overview of the connections between studied variables and theoretical frameworks.

Study Variables	JD–R Model	DISC Model	TTS
Time pressure	X		
Emotional demands	X	X	
Physical demands	X	X	
Role ambiguity	X		
Autonomy	X		
Social support	X		
Physical resources	X	X	
Participation in DM	X		
Challenge appraisal			X
Threat appraisal			X

Note: JD–R = Job Demands–Resources Model; DISC = Demand-Induced Strain Compensation model; TTS = Transactional Theory of Stress.

**Table 2:** Fit statistics for the measurement models.

Model	χ^2^			RMSEA		SRMR	CFI	TLI
	Value	*df*	*p*	Value	90%CI			
Time pressure	62.319	60	0.394	0.010	[0.000;0.032]	0.026	0.998	0.999
Emotional demands	71.988	49	0.018	0.035	[0.015;0.052]	0.035	0.989	0.985
Physical demands	76.206	59	0.065	0.027	[0.000;0.042]	0.042	0.993	0.991
Role ambiguity	74.212	38	0.000	0.050	[0.033;0.067]	0.029	0.982	0.973

Note: MLR estimator. RMSEA = Root Mean Square Error of Approximation, SRMR = Standardized Root Mean Square Residuals, CFI = Comparative Fit Index, TLI = Tucker–Lewis Index.

**Table 3:** Means, standard deviations, reliabilities (Omega), and correlations for study variables.

Variable	*M*	*SD*	1	2	3	4	5	6	7	8	9	10	11	12	13	14	15	16
1. TP	3.85	0.75	(0.75)															
2. TPCA	2.28	0.89	−0.14 **	(0.77)														
3. TPTA	3.49	1.00	0.40 ***	−0.48 ***	(0.85)													
4. ED	3.93	0.85	0.33 ***	−0.13 ***	0.29 ***	(0.78)												
5. EDCA	2.71	0.93	−0.19 ***	0.45 ***	−0.32 ***	−0.07 ^+^	(0.81)											
6. EDTA	2.75	1.10	0.28 ***	−0.23 ***	0.54 ***	0.29 ***	−0.38 ***	(0.87)										
7. PD	3.73	1.09	0.26 ***	−0.09 *	0.18 ***	0.18 ***	−0.01	0.13 **	(0.93)									
8. PDCA	2.00	0.90	−0.08	0.38 ***	−0.16 ***	−0.09 *	0.47 ***	−0.06	0.08	(0.80)								
9. PDTA	3.35	1.20	0.23 ***	−0.30 ***	0.47 ***	0.26 ***	−0.20 ***	0.42 ***	0.38 ***	−0.21 ***	(0.90)							
10. RA	2.41	0.94	0.16 **	−0.06	0.20 ***	0.14 ***	−0.13 *	0.19 ***	0.02	0.01	0.14 **	(0.85)						
11. RACA	2.68	1.00	−0.11 *	0.34 ***	−0.26 ***	−0.09	0.30 ***	−0.09	0.02	0.28 ***	−0.15 **	−0.20 ***	(0.85)					
12. RATA	3.10	1.14	0.21 ***	−0.23 ***	0.43 ***	0.23 ***	−0.15**	0.39 ***	0.07	−0.07	0.40 ***	0.25 ***	−0.53 ***	(0.90)				
13. AU	2.60	0.88	−0.32 ***	0.14**	−0.23 ***	−0.16 ***	0.18 ***	−0.13 **	−0.30 ***	0.10 *	−0.19 ***	−0.22 ***	0.08	−0.12 *	(0.80)			
14. SS	3.81	0.92	−0.01	0.01	−0.07	−0.09 *	0.13 **	−0.10 *	0.07	−0.01	0.02	−0.24 ***	0.07	−0.08	0.14 **	(0.90)		
15. PR	2.85	0.79	−0.28 ***	0.13 ***	−0.22 ***	−0.23 ***	0.22 ***	−0.13 **	−0.12 **	0.17 ***	−0.17 ***	−0.17 ***	0.09	−0.16**	0.36 ***	0.19 ***	(0.69)	
16. PA	2.64	1.03	−0.22 ***	0.18 ***	−0.26 ***	−0.20 ***	0.20 ***	−0.20 ***	−0.16 ***	0.16 ***	−0.23 ***	−0.27 ***	0.18 ***	−0.27 ***	0.57 ***	0.23 ***	0.35 ***	(0.80)

Note: TP = Time Pressure; TPCA = Challenge Appraisal of Time Pressure; TPTA = Threat Appraisal of Time Pressure; ED = Emotional Demands; EDCA = Challenge Appraisal of Emotional Demands; EDTA = Threat Appraisal of Emotional Demands; PD = Physical Demands; PDCA = Challenge Appraisal of Physical Demands; PDTA = Threat Appraisal of Physical Demands; RA = Role Ambiguity; RACA = Challenge Appraisal of Role Ambiguity; RATA = Threat Appraisal of Role Ambiguity; AU = Autonomy; SS = Social Support; PR = Physical Resources; PA = Participation in Decision-making. Coefficients display zero-order correlations and internal consistencies (McDonald’s ω) in the main diagonal. ^+^ *p* = 0.05. * *p* < 0.05. ** *p* < 0.01. *** *p* < 0.001.

**Table 4:** Linear model of predictors of nurses’ challenge and threat appraisal of time pressure.

	Challenge Appraisal				Threat Appraisal			
	β	*SE*	95%CI	*p*	β	*SE*	95%CI	*p*
Model #1								
Constant	−0.01	0.05	[−0.11, 0.09]	0.882	0.01	0.05	[−0.08, 0.10]	0.874
Time pressure	−0.11 ^+^	0.05	[−0.22, −0.01]	0.034	0.36 **	0.05	[0.26, 0.45]	0.001
Autonomy	0.12 *	0.05	[0.02, 0.22]	0.021	−0.12 *	0.05	[−0.21, −0.02]	0.016
Time pressure × Autonomy	0.01	0.05	[−0.09, 0.09]	0.995	−0.01	0.04	[−0.09, 0.08]	0.921

Note: ^+^ *p* < 0.05. * *p* < 0.025. ** *p* < 0.010.

**Table 5:** Linear model of predictors of nurses’ challenge and threat appraisal of emotional demands.

	Challenge Appraisal				Threat Appraisal			
	β	*SE*	95%CI	*p*	β	*SE*	95%CI	*p*
Model #1								
Constant	−0.01	0.05	[−0.11, 0.09]	0.877	0.00	0.05	[−0.09, 0.09]	0.998
Emotional demands	−0.07	0.05	[−0.17, 0.03]	0.151	0.31 ***	0.05	[0.21, 0.40]	<0.001
Social support	13 *	0.05	[0.03, 0.23]	0.011	−0.07	0.05	[−0.16, 0.03]	0.180
Emotional demands × Social support	−0.01	0.06	[−0.12, 0.10]	0.816	−0.02	0.05	[−0.12, 0.09]	0.775

Note: * *p* < 0.025. *** *p* < 0.001.

**Table 6:** Linear model of predictors of nurses’ challenge and threat appraisal of physical demands.

	Challenge Appraisal				Threat Appraisal			
	β	*SE*	95%CI	*p*	β	*SE*	95%CI	*p*
Model #1								
Constant	0.01	0.05	[−0.09, 0.10]	0.895	0.00	0.05	[−0.09, 0.09]	0.990
Physical demands	0.10	0.05	[−0.01, 0.20]	0.056	0.38 ***	0.05	[0.29, 0.47]	<0.001
Physical resources	0.21 ***	0.05	[0.11, 0.30]	<0.001	−0.14 **	0.05	[−0.23, −0.05]	0.002
Physical demands × Physical resources	0.0	0.04	[−0.05, 0.12]	0.389	−0.05	0.04	[−0.13, 0.03]	0.250

Note: ** *p* < 0.010. *** *p* < 0.001.

**Table 7:** Linear model of predictors of nurses’ challenge and threat appraisal of role ambiguity.

	Challenge Appraisal				Threat Appraisal			
	β	*SE*	95%CI	*p*	β	*SE*	95%CI	*p*
Model #1								
Constant	−0.01	0.05	[−0.10, 0.10]	0.983	−0.01	0.05	[−0.11, 0.09]	0.847
Role ambiguity	−0.16 **	0.05	[−0.26, −0.06]	0.002	0.19 ***	0.05	[0.09, 0.29]	<0.001
Participation in decision making	0.14 **	0.05	[0.04, 0.24]	0.007	−0.21 ***	0.05	[−0.30, −0.11]	<0.001
Role ambiguity × Participation in decision making	−0.01	0.05	[−0.10, 0.08]	0.903	−0.02	0.04	[−0.11, 0.06]	0.592

Note: ** *p* < 0.010. *** *p* < 0.001.

## Data Availability

The data that support the findings of this study are available from the corresponding author upon reasonable request.

## Supplementary Materials

The following supporting information can be downloaded at: https://www.mdpi.com/article/10.3390/ijerph20021288/s1, Code S1: R Code for data analysis.
