# Supplementary material for: Predicting Challenge and Threat Appraisal of Job Demands among Nurses: The Role of Matching Job Resources
Source: Int J Environ Res Public Health. 2023 Jan 11;20(2):1288. doi: 10.3390/ijerph20021288 (PMC9859003; doi:10.3390/ijerph20021288)
Supplement: Supplementary file 1 [file ijerph-20-01288-s001.zip › ijerph-2045486-supplementary.pdf]

Supplemental material (S1): R Code for data analysis

R Code for the descriptive statistics and correlational analyses

Preliminary steps

```
#Load Data set
load("../data/LetzCare02_var.RData")

#Load required package
library(psych)

#Disable abbreviated results
options(scipen=999)
```

Descriptive statistics

```
#select variables
Stud.variables <- data.frame(LetzCare02.var$TP,LetzCare02.var$TPCA,LetzCare02
.var$TPTA,LetzCare02.var$ED,LetzCare02.var$EDCA,LetzCare02.var$EDTA,LetzCare0
2.var$PD,LetzCare02.var$PDCA,LetzCare02.var$PDTA,LetzCare02.var$RA,LetzCare02
.var$RACA,LetzCare02.var$RATA,LetzCare02.var$AU,LetzCare02.var$SS,LetzCare02.
var$PR,LetzCare02.var$PA)

#descriptive statistics
des.Stud.var <- describe(Stud.variables)

#display descriptive statistics
des.Stud.var

#correlations
corel.Stud.var <- corr.test(Stud.variables, use="complete")

#create table
table <-cbind(M = des.Stud.var$mean, SD = des.Stud.var$sd, corel.Stud.var$r)

#display table
table

#export table
write.csv(table, file = "../outputs/DescriptiveStatistics.csv")
```

R Code for the Factor Analyses

Preliminary Steps

```
#Load Data set
load("../data/LetzCare02_trans.RData")

#Load package
library(lavaan)

#Activate Data set
attach(LetzCare02.trans)
```

Time Pressure

```
#Specify the model
cfa.TP <- '
           #Measurement model
           TP =~ v1*TP01 + v1*TP02
           TPCA =~ TPCA01 + TPCA02 + TPCA03 + TPCA04
           TPTA =~ TPTA01 + TPTA02 + TPTA03
           AU =~ AU01 + AU02 + AU03 + AU04'

#Fit the model
fit.TP <- cfa(cfa.TP, data = LetzCare02.trans, estimator = "MLR", missing ="f
iml")

#Display summary output
summary(fit.TP, fit = TRUE, standardized = TRUE, rsquare = TRUE)
```

Emotional demands

```
#Specify the model
cfa.ED <- '
          #Measurement model
           ED =~ v1*ED01 + v1*ED02
           EDCA =~ EDCA01 + EDCA02 + EDCA03 + EDCA04
           EDTA =~ EDTA01 + EDTA02 + EDTA03
           SS =~ SS01 + SS02 + SS03'

#Fit the model
fit.ED <- cfa(cfa.ED, data = LetzCare02.trans, estimator = "MLR", missing = "
fiml")

#Display summary output
summary(fit.ED, fit = TRUE, standardized = TRUE, rsquare = TRUE)
```

Physical demands

```
#Specify the model
cfa.PD <- '
           #Measurement model
           PD =~ PD01 + PD02 + PD03
           PDCA =~ PDCA01 + PDCA02 + PDCA03 + PDCA04
           PDTA =~ PDTA01 + PDTA02 + PDTA03
           PR =~ PR01 + PR02 + PR03'

#Fit the model
fit.PD <- cfa(cfa.PD, data = LetzCare02.trans, estimator = "MLR", missing = "
fiml")

#Display summary output
summary(fit.PD, fit = TRUE, standardized = TRUE, rsquare = TRUE)
```

Role ambiguity

```
#Specify the model
cfa.RA <- '
           #Measurement model
           RA =~ RA01r + RA02r
           RACA =~ RACA01 + RACA02 + RACA03 + RACA04
           RATA =~ RATA01 + RATA02 + RATA03
           PA =~ PA01 + PA02'

#Fit the model
fit.RA <- cfa(cfa.RA, data = LetzCare02.trans, estimator = "MLR", missing = "
fiml")

#Display summary output
summary(fit.RA, fit = TRUE, standardized = TRUE, rsquare = TRUE)
```

R Code for Regression Analyses (Simple Moderation)

Time pressure

1.1.Preliminary steps

```
#Load Data set
load("../data/LetzCare02_var.RData")

#View Data set
View(LetzCare02.var)

#Center predictor variables
LetzCare02.var$TP_cen <- as.numeric(scale(LetzCare02.var$TP, scale = FALSE, c
enter = TRUE))

LetzCare02.var$AU_cen <- as.numeric(scale(LetzCare02.var$AU, scale = FALSE, c
enter = TRUE))

#View Data set
View(LetzCare02.var)

#Disable abbreviated results
options(scipen=999)
```

1.2.Dependent variable: Challenge appraisal

```
#Activate Data set
attach(LetzCare02.var)

#Specify model, calculate standardized regression estimates
mod.reg.TPCA <-lm(scale(TPCA) ~scale(TP_cen)*scale(AU_cen))

#Display output
summary(mod.reg.TPCA)

#Display 95% CI
confint(mod.reg.TPCA)
```

1.3.Dependent variable: Threat appraisal

```
#Specify model, calculate standardized regression estimates
mod.reg.TPTA <-lm(scale(TPTA) ~scale(TP_cen)*scale(AU_cen))

#Display output
summary(mod.reg.TPTA)

#Display 95% CI
confint(mod.reg.TPTA)
```

Emotional demands

2.1. Preliminary steps

```
#Load Data set
load("../data/LetzCare02_var.RData")

#View Data set
View(LetzCare02.var)

#Center predictor variables
LetzCare02.var$ED_cen <- as.numeric(scale(LetzCare02.var$ED, scale = FALSE, c
enter = TRUE))

LetzCare02.var$SS_cen <- as.numeric(scale(LetzCare02.var$SS, scale = FALSE, c
enter = TRUE))

#View Data set
View(LetzCare02.var)

#Disable abbreviated results
options(scipen=999)
```

2.2. Dependent variable: Challenge Appraisal

```
#Activate Data set
attach(LetzCare02.var)

#Specify model, calculate standardized regression estimates
mod.reg.EDCA <-lm(scale(EDCA) ~scale(ED_cen)*scale(SS_cen))

#Display output
summary(mod.reg.EDCA)

#Display 95% CI
confint(mod.reg.EDCA)
```

2.3. Dependent variable: Threat appraisal

```
#Specify model, calculate standardized regression estimates
mod.reg.EDTA <-lm(scale(EDTA) ~scale(ED_cen)*scale(SS_cen))

#Display output
summary(mod.reg.EDTA)

#Display 95% CI
confint(mod.reg.EDTA)
```

Physical demands

3.1. Preliminary analyses

```
#Load Data set
load("../data/LetzCare02_var.RData")

#View Data set
View(LetzCare02.var)

#Center predictor variables
LetzCare02.var$PD_cen <- as.numeric(scale(LetzCare02.var$PD, scale = FALSE, c
enter = TRUE))

LetzCare02.var$PR_cen <- as.numeric(scale(LetzCare02.var$PR, scale = FALSE, c
enter = TRUE))

#View Data set
View(LetzCare02.var)

#Disable abbreviated results
options(scipen=999)
```

3.2. Dependent variable: Challenge appraisal

```
#Activate Data set
attach(LetzCare02.var)

#Specify model, calculate standardized regression estimates
mod.reg.PDCA <-lm(scale(PDCA) ~scale(PD_cen)*scale(PR_cen))

#Display output
summary(mod.reg.PDCA)

#Display 95% CI
confint(mod.reg.PDCA)
```

3.3. Dependent variable: Threat appraisal

```
#Specify model, calculate standardized regression estimates
mod.reg.PDTA <-lm(scale(PDTA) ~scale(PD_cen)*scale(PR_cen))

#Display output
summary(mod.reg.PDTA)

#Display 95% CI
confint(mod.reg.PDTA)
```

Role ambiguity

4.1. Preliminary analyses

```
#Load Data set
load("../data/LetzCare02_var.RData")

#View Data set
View(LetzCare02.var)

#Center predictor variables
LetzCare02.var$RA_cen <- as.numeric(scale(LetzCare02.var$RA, scale = FALSE, c
enter = TRUE))

LetzCare02.var$PA_cen <- as.numeric(scale(LetzCare02.var$PA, scale = FALSE, c
enter = TRUE))

#View Data set
View(LetzCare02.var)

#Disable abbreviated results
options(scipen=999)
```

4.2.Dependent variable: Challenge appraisal

```
#Activate Data set
attach(LetzCare02.var)

#Specify model, calculate standardized regression estimates
mod.reg.RACA <-lm(scale(RACA) ~scale(RA_cen)*scale(PA_cen))

#Display output
summary(mod.reg.RACA)

#Display 95% CI
confint(mod.reg.RACA)
```

4.3.. Dependent variable: Threat appraisal

```
#Specify model, calculate standardized regression estimates
mod.reg.RATA <-lm(scale(RATA) ~scale(RA_cen)*scale(PA_cen))

#Display output
summary(mod.reg.RATA)

#Display 95% CI
confint(mod.reg.RATA)
```
